# The COVID-19 pandemic should not jeopardize dengue control

**DOI:** 10.1371/journal.pntd.0008716

**Published:** 2020-09-23

**Authors:** Marie-Marie Olive, Thierry Baldet, James Devillers, Johanna Fite, Marie-Claire Paty, Christophe Paupy, Philippe Quénel, Elsa Quillery, Jocelyn Raude, Jean-Paul Stahl, Marie Thiann-Bo-Morel, David Roiz

**Affiliations:** 1 MIVEGEC, Univ. Montpellier, IRD, CNRS, Montpellier, France; 2 ASTRE, Univ. Montpellier, Cirad, INRA, Sainte-Clotilde, La Réunion, France; 3 CTIS, Rillieux La Pape, France; 4 ANSES, Maison Alfort, France; 5 Santé publique France (French National Public Health Agency), Saint-Maurice, France; 6 Univ Rennes, Inserm, EHESP, Irset-UMR_S 1085, Rennes, France; 7 EHESP School of Public Health, Rennes, France; 8 CHUGA and Grenoble Alpes University, Grenoble, France; 9 Espace-Dev (UMR 228), Faculté des Sciences de l’Homme et de l’Environnement, Université de La Réunion, Le Tampon, La Réunion, France; University of Florida, UNITED STATES

## Abstract

The concurrent circulation of dengue and coronavirus disease 2019 (COVID-19) may produce many unfavourable outcomes—such as co-infections; delays in diagnosis, treatment, and mitigation measures; overwhelming of the healthcare system; underreporting of cases; deterioration in surveillance and control interventions; and exacerbation of social inequalities. Indeed, lockdown is greatly compromising the effectiveness of vector control, especially social mobilization campaigns and preventive insecticide spraying in private spaces (indoor and peridomestic spraying). Thus, failure to appropriately implement the full range of vector control interventions can lead to a reduction in their overall effectiveness and an increasing risk of vector-borne diseases circulating. Consequently, the health community and policy makers should develop proactive policies and allocate adequate resources to prevent and manage the resurgence of dengue and other vector-borne diseases in the new era of COVID-19.

## Viewpoints

**Fig 1:**
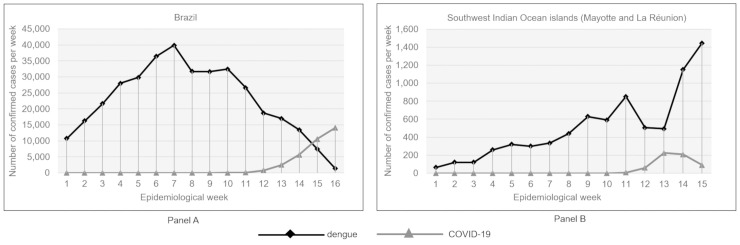
Confirmed cases of COVID-19 and dengue in Brazil (Panel A) and the Southwest Indian Ocean islands (La Réunion and Mayotte; Panel B) [5–9]. COVID-19, coronavirus disease 2019.

The coronavirus disease 2019 (COVID-19) pandemic, caused by the emerging severe acute respiratory syndrome coronavirus 2 (SARS-CoV-2), is now a major health crisis. By mid July 2020, there had been about 14 million cases and more than 600,000 deaths worldwide, and there have been massive economic consequences and social disruption. While this global crisis rightly demands the world’s attention, many other infectious diseases are still on the rise and risk increasing further as the focus shifts away from them. This is the case with dengue fever, a viral disease transmitted by *Aedes* mosquitoes, whose incidence has increased dramatically over the past decade [1], with severe epidemics currently affecting Latin America, Asia, and the Indian Ocean [2–6]. Brazil, for example, is currently experiencing the largest ever dengue epidemic, with nearly 800,000 cases and 221 deaths between January and April 2020 [3,7] (Fig 1, panel A). Similarly, Southwest Indian Ocean islands, such as La Réunion and Mayotte (two French overseas departments), are experiencing unprecedented dengue outbreaks, with more than 30,000 cases reported on the islands since 2017 [5,6] (Fig 1, panel B). At the same time, other arboviruses also transmitted by *Aedes* mosquitoes, such as chikungunya, Zika, and yellow fever, continue to occur in many parts of the world [10], and temperate regions of the Northern Hemisphere (United States and Europe), which are particularly affected by the COVID-19 pandemic, are also threatened by autochthonous transmission of dengue, chikungunya, and Zika in areas where the vectors *A*. *aegypti* and/or *A*. *albopictus* are established [11].

In April 2020, in a context of concurrent epidemics of dengue and COVID-19 in the French overseas departments, the French Ministry of Health tasked the French Agency for Food, Environmental and Occupational Health & Safety (ANSES) with setting up a multidisciplinary task force of experts to assess the impact of the COVID-19 pandemic and lockdown (imposed from 17 March to 11 May in France) on dengue surveillance and vector control interventions. Their report was recently published [12], and in this letter, the task force presents the results of its expert assessment and its recommendations to the global health community.

## Considerations and recommendations

Concurrent circulation of COVID-19 and dengue can lead to a delay in diagnosis and may therefore negatively affect the management, care, and control interventions specific to each of the 2 diseases. Differential diagnosis is required because the 2 viral infections share certain clinical features, such as fever, fatigue, and headache [4,13–16]. In addition, concern has been raised over the discovery of cases of false-positive dengue serology in individuals with COVID-19 [17]. Confusion in diagnosis or in test results can have dramatic consequences not only for patients but also for public health control interventions (e.g., contact tracing in the case of COVID-19 and vector control in the case of dengue). Co-circulation of the 2 viruses in the same regions exacerbates the health risk because of the potentially critical consequences of both infections in terms of morbidity and mortality, especially in severe cases. Although co-infection of dengue and COVID-19 has so far been only sporadically documented in Thailand, Singapore, Mayotte, and La Réunion islands [16,18], it is raising serious concerns in countries affected by dengue outbreaks, particularly in Southeast Asia and South America [2–4, 13–20]. Healthcare services in many countries have already been disrupted or even overwhelmed by the COVID-19 pandemic, and co-occurrence of the 2 diseases often aggravates the situation. This is particularly worrying in regions where several dengue serotypes are co-circulating and where secondary or even tertiary infections have been recorded [5,7]. Epidemiological surveillance of dengue has also been detrimentally affected. In all the French overseas departments, there was a drop in declared notified dengue cases just after the start of the lockdown (epidemiological weeks 12–13, Fig 1, panel B), even though there had been a marked increase in the number of cases over the previous weeks [12]. Underreporting of dengue, notable for paucisymptomatic cases, can be attributed not only to lockdown and to difficulties in moving around, even for medical reasons, but also to public concern over the risk of being infected by COVID-19 in health facilities, the closure of some clinics, and disrupted access to diagnosis [12]. The COVID-19 pandemic also affects vector control of *Aedes* mosquitoes in drawing attention and collective mobilization away from dengue and other arboviral diseases and their vector mosquitoes. In the wake of the COVID-19 crisis and lockdown, vector control interventions were scaled down in all the French overseas departments, social mobilization campaigns were put on hold, and preventive insecticide spraying was curtailed, especially in private spaces (indoor and peridomestic spraying) [12]. In the absence of vaccines and therapeutic treatment, the prevention and control of dengue and other *Aedes*-borne viral diseases continues to rely heavily on controlling mosquito vector populations or on interrupting human–vector contact through integrated, sustainable, synergic, and proactive vector management [1,21]. Failure to appropriately implement the full range of vector control interventions can reduce their overall effectiveness [1,21]. In fact, many of the interventions that are an essential part of an effective dengue control programme targeting private properties—such as indoor residual spraying, door-to-door campaigning, source reduction, and peridomestic residual spraying (Table 1)—are at odds with the guidelines for COVID-19 prevention and lockdown, simply because they require or may entail close contact between the vector control teams and the resident population. As such, interventions forming the core of efficient dengue control are expected to be significantly scaled down in many areas of the world where they are commonly carried out [1,12]. In an effort to fill this gap, while acknowledging the effectiveness of social mobilization in the control and prevention of *Aedes*-borne diseases [1,21], we consider it is crucial to strengthen communication for social mobilization against these diseases using digital communication tools and social networks jointly with the dissemination of information concerning COVID-19 (e.g., eliminating mosquito breeding sites while staying home), and to adapt existing guidelines to meet the current situation (Table 1) [19, 20]. Thus, vector control workers should use personal protective equipment and physical distancing while carrying out any vector control interventions, including those targeting larvae and adult mosquitoes, as well as community-based control measures (Table 1) [12,19,20]. In addition, local teams that know how to make information more effective in the field [20] could combine messages aimed at strengthening household and community participation in vector control measures with COVID-19 prevention and best practice messages [19,20]. Consequently, it is essential to reaffirm the crucial role of vector control staff, not only for dengue control measures but also to leverage community ownership and engagement in public health, and in particular to ensure the safety of these agents facing COVID-19, so that they can guarantee the safety of populations confronted with dengue fever.

**Table 1:** Effectiveness, strength of evidence of vector control measures [1, 12], assessment of the risk of transmission of COVID-19 for vector control workers, and recommendation for maintaining vector control measures in compliance with COVID-19 prevention (table adapted from the task force report [12] and updated [19, 20]).

Vector control measures	Effectiveness [1,12,19]	Strength of evidence [1, 12]	Type of transmission risk of COVID-19	Degree of transmission risk of COVID-19 assessed by the expert group	Recommendations
**Interventions in public areas**					
**Outdoor spatial spraying with vehicle mounted**	Low	Low	Contact with colleague within vehicle	Low	One person per vehicle or staggered seating and wearing masks Vehicle ventilation (between each use)
			Contact with fomites within vehicle	Low	A vehicle assigned to a given driver during the journey Hand washing (soap, hydroalcoholic gel) Regular cleaning of surfaces in contact with hands (e.g., door handles, steering wheel, gear lever, armrests, etc.) Establish a protocol for cleaning vehicles after each use
**Outdoor spatial spraying with portable equipment**	Moderate	Low	Contact with public	Moderate	Physical distancing (>1 m) Wearing mask/protective visor Reduction of contact time with people
			Contact with fomites	Low	Hand washing (soap, hydroalcoholic gel) Wearing disposable gloves
**Eliminating nonpermanent breeding sites**	High	Moderate	Contact with public	Moderate	Physical distancing (>1 m) Wearing mask/protective visor Reduction of contact time with people The elimination of breeding sites must be carried out without any exchange or proximity with the resident(s) of the house visited During lockdown, households should be encouraged to work together in and around their homes to get rid of stagnant water, reduce solid wastes, and ensure proper covering of all water storage containers
			Contact with fomites	Moderate	Hand washing (soap, hydroalcoholic gel) Wearing disposable gloves
**Larviciding**	High	Moderate	Contact with public	Moderate	Physical distancing (>1 m) Wearing mask/protective visor Reduction of contact time with people
			Contact with fomites	Low	Hand washing (soap, hydroalcoholic gel) Wearing disposable gloves
**Interventions in private areas (houses)**					
**Eliminating nonpermanent breeding sites**	Moderate	Low	Contact with residents	High	Physical distancing (>1 m) Wearing mask/protective visor Reduction of contact time with people The elimination of breeding sites must be carried out without any exchange or proximity with the resident(s) of the house visited During lockdown, households should be encouraged to work together in and around their homes to get rid of stagnant water, reduce solid wastes, and ensure proper covering of all water storage containers
			Contact with fomites	High	Hand washing (soap, hydroalcoholic gel) Wearing disposable gloves
**Intradomiciliar treatments**	High	High	Contact with residents	High	Physical distancing (>1 m) Wearing mask/protective visor Reduction of contact time with people
			Contact with fomites	High	Hand washing (soap, hydroalcoholic gel) Wearing disposable gloves
**Peridomiciliar treatments**	Moderate	Moderate	Contact with residents	High	Physical distancing (>1 m) Wearing mask/protective visor Reduction of contact time with people
			Contact with fomites	Moderate	Hand washing (soap, hydroalcoholic gel) Wearing disposable gloves
**Social mobilization**					
**Social mobilization: door to door**	High	High	Contact with residents	High	Physical distancing (>1 m) Wearing mask/protective visor Reduction of contact time with people Under the present situation, even during lockdown, households should be encouraged to work together in and around their homes to get rid of stagnant water, reduce solid wastes, and ensure proper covering of all water storage containers
			Contact with fomites	Moderate	Hand washing (soap, hydroalcoholic gel) Wearing disposable gloves
**Social mobilization during event gathering people**	Moderate	Low to moderate	Contact with residents	High	The organisation of this type of event must be considered in the light of COVID-19 epidemiological situation Excluded during lockdown and/or during COVID-19 high circulation In areas where schools are reopened, special sessions should be devoted to awareness of COVID-19 and dengue prevention (including vector control measures) Organise the event outdoor Physical distancing (>1 m) Wearing mask/protective visor Reduction of contact time with people Limit the number of persons
			Contact with fomites	High	Hand washing (soap, hydroalcoholic gel) Wearing disposable gloves
**Social mobilization (media, social networks, etc.)**	Moderate	Low to moderate	No contact	Null	No restrictions Joint communication of prevention messages for COVID-19 and dengue prevention (including vector control measures)
**Within work premises**					
**Within the work premises**	NA	NA	Contact with colleagues	High	Physical distancing (>1 m) Shifting schedule offset Team partitioning Limiting team size Limiting time spent in premises Wearing mask/protective visor
			Contact with fomites	High	Hand washing (soap, hydroalcoholic gel) Ensure regular cleaning of the premises (handles, furniture, washbasins, etc.) and increase its frequency (adapt the cleaning protocol)

**Abbreviations:** COVID-19, coronavirus disease 2019; NA, not applicable

The negative impact of the COVID-19 crisis on dengue surveillance and control is obvious in tropical areas affected by dengue epidemics, but the risk of local cases of dengue and other arboviruses in temperate regions is expected to be lower as travel restrictions limit the opportunities for viral importation. However, the risk is still there, as 14 dengue cases were imported into European France during the lockdown period from 17 March to 11 May 2020, despite the very low number of flights returning from dengue-affected areas [12]. The resumption, even partial, of air transport this summer will bring with it a risk of viraemic travellers returning from regions affected by these arboviruses during the vector season in Europe [11,12]. Neglecting early vector control intervention around imported cases may also increase the risk of autochthonous cases of arbovirus [11]. Finally, the adverse economic and social consequences could be aggravated by concurrent circulation of these diseases. Social inequalities in relation to dengue and other arboviruses, such as Zika, have been highlighted [22]. Similarly, it has recently emerged that the COVID-19 pandemic is exacerbating social inequalities, as has been shown in the French overseas departments, such as La Réunion and Mayotte, as well as in the US [23,24].

## Conclusion

Public health systems are faced with the challenge of co-circulation of dengue and COVID-19 and the many detrimental outcomes—which include co-infections; delays in diagnosis, treatment, and mitigation measures; overwhelming of healthcare systems; underreporting of cases; deterioration of surveillance and vector control interventions; and exacerbation of social inequalities. If the effectiveness of vector control measures is compromised, this can have serious consequences for public health. We have therefore put forward a framework for dealing with these risks on a global level (Table 1) [12]. As highlighted earlier [19,20], we have drawn attention to the importance of maintaining and strengthening integrated management of mosquito-borne diseases—especially surveillance, vector control in private spaces (door-to-door campaigns, indoor and peridomestic sprayings) and social mobilization—during the COVID-19 pandemic, and to the importance of upgrading guidance to take into account the need to minimise workers and citizens being exposed to risk. Even as several countries are unlocked, there is wide concern over the possibility of a second wave of the COVID-19 epidemic, and a new (strict or partial) lockdown is underway in some regions [25]. Throughout the world, we are facing an imminent potential risk of arboviral diseases, such as dengue fever, chikungunya, Zika, and yellow fever, surging simultaneously due to a deterioration in surveillance and vector control and giving rise to a more severe situation than the COVID-19 pandemic alone. We urge the health community and policy makers to urgently develop proactive policies and allocate adequate resources to prevent and manage the resurgence of vector-borne diseases in the new era of COVID-19. More broadly, in the context of the risk of new emerging disease occurrence with high epidemic and pandemic potential, advance preparedness will be essential to maintain and strengthen vector-borne disease control activities.
